# 2-(7-Meth­oxy-1-naphth­yl)acetonitrile

**DOI:** 10.1107/S1600536810024372

**Published:** 2010-06-30

**Authors:** Wen-bin Wei, Ru Jia, Jie Sun, Hai-Bo Wang

**Affiliations:** a College of Food Science and Light Industry, Nanjing University of Technology, Xinmofan Road No.5 Nanjing, Nanjing 210009, People’s Republic of China; b College of Science, Nanjing University of Technology, Xinmofan Road No.5 Nanjing, Nanjing 210009, People’s Republic of China

## Abstract

The mol­ecule of the title compound, C_13_H_11_NO, is almost planar (r.m.s. deviation = 0.013 Å), apart from the cyanide group, for which the C and N atoms deviate from the mean plane of the other atoms by 0.341 (3) and 0.571 (4) Å, respectively. In the crystal, weak aromatic π–π stacking [centroid–centroid distance = 3.758 (3) Å] may help to stabilize the structure.

## Related literature

For background to the use of naphthyl­ethyl acetonitrile as an inter­mediate for the synthesisis of *N*-naphthyl­ethyl amide derivatives, see: Depreux & Lesieur (1994 ▶). For further synthetic details, see: Yous & Andrieux (1992 ▶).
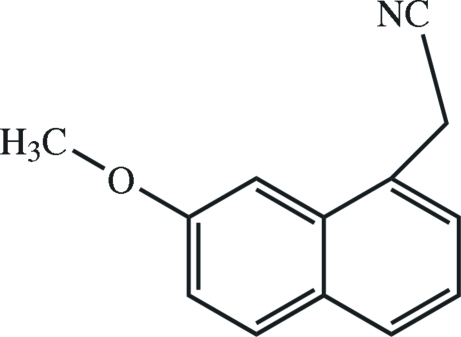


## Experimental

### 

#### Crystal data

C_13_H_11_NO
*M* *_r_* = 197.23
Monoclinic, 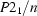

*a* = 7.5110 (15) Å
*b* = 9.6170 (19) Å
*c* = 14.731 (3) Å
β = 101.03 (3)°
*V* = 1044.4 (4) Å^3^
*Z* = 4
Mo *K*α radiation
μ = 0.08 mm^−1^
*T* = 293 K
0.30 × 0.20 × 0.10 mm

#### Data collection

Enraf–Nonius CAD-4 diffractometer
Absorption correction: ψ scan (North *et al.*, 1968 ▶) *T*_min_ = 0.976, *T*_max_ = 0.992
1971 measured reflections
1897 independent reflections
1045 reflections with *I* > 2σ(*I*)
*R*_int_ = 0.011
3 standard reflections every 200 reflections  intensity decay: 1%

#### Refinement

*R*[*F* ^2^ > 2σ(*F* ^2^)] = 0.059
*wR*(*F* ^2^) = 0.163
*S* = 1.00
1897 reflections
136 parameters
H-atom parameters constrained
Δρ_max_ = 0.14 e Å^−3^
Δρ_min_ = −0.14 e Å^−3^

### 

Data collection: *CAD-4 EXPRESS* (Enraf–Nonius, 1994 ▶); cell refinement: *CAD-4 EXPRESS*; data reduction: *XCAD4* (Harms & Wocadlo, 1995 ▶); program(s) used to solve structure: *SHELXS97* (Sheldrick, 2008 ▶); program(s) used to refine structure: *SHELXL97* (Sheldrick, 2008 ▶); molecular graphics: *PLATON* (Spek, 2009 ▶); software used to prepare material for publication: *PLATON*.

## Supplementary Material

Crystal structure: contains datablocks global, I. DOI: 10.1107/S1600536810024372/hb5505sup1.cif

Structure factors: contains datablocks I. DOI: 10.1107/S1600536810024372/hb5505Isup2.hkl

Additional supplementary materials:  crystallographic information; 3D view; checkCIF report

## Figures and Tables

**Fig. 1.**
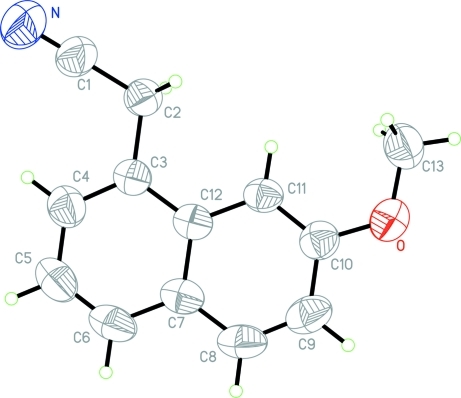
View of the title compound with displacement ellipsoids for non-H atoms drawn at the 50% probability level.

**Fig. 2.**
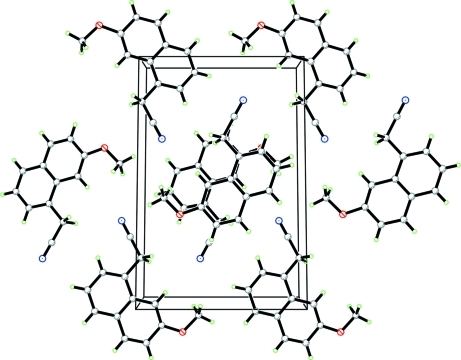
A packing diagram of title molecule.

## supplementary crystallographic information

### Comment

Naphthylethyl acetonitrile is an important pharmaceutical intermediate for synthesizing *N*-naphthylethyl amide derivatives which was evaluated as melatonin receptor ligands (Depreux & Lesieur, 1994). We report herein the crystal structure of the title compound, (I).

In the molecule of the title compound (Fig 1), the bond lengths and angles are within normal ranges. Rings A (C3—C7/C12), B (C8—C12)are, of course, planar, and they are oriented at dihedral angle A/B= 1.10 (3) °. So, they are nearly coplanar. No classical hydrogen bond was found in the molecule. The π–π contacts between the naphthalene rings, *Cg*1—*Cg*2^i^ [symmetry codes: -*x*,1 - *y*,1 - *z*, where *Cg*1 and *Cg*2 are centroids of the rings A (C3—C7/C10/C12), and B (C8—C12), respectively] may further stabilize the structure, with centroid-centroid distances of 3.758 (3) Å.

### Experimental

(7-Methoxy-1-naphthyl)acetic acid was reacted with thionyl chloride in CHCl_3_, and the crude acid chloride was treated with aqueous ammonia to produce (7-Methoxy-1-naphthyl)acetamide. Dehydration of this amide with trifluoroacetic anhydride in THF at 273 K gave the title compound (Yous & Andrieux, 1992). Crystals suitable for X-ray analysis were obtained by slow evaporation of a methanol solution (yield; 66%, m.p. 353 K).

### Refinement

H atoms were positioned geometrically, with C—H = 0.93 Å for aromatic H and C—H = 0.96 and 0.97 Å for methyl and methylene H, respectively, and constrained to ride on their parent atoms, with *U*_iso_(H) = *xU*_eq_(C,*O*), where *x* = 1.5 for OH H and *x* = 1.2 for all other H atoms.

### Crystal data

**Table:**

C_13_H_11_NO	*F*(000) = 416
*M**_r_* = 197.23	*D*_x_ = 1.254 Mg m^−^^3^
Monoclinic, *P*2_1_/*n*	Melting point: 353 K
Hall symbol: -P 2yn	Mo *K*α radiation, λ = 0.71073 Å
*a* = 7.5110 (15) Å	Cell parameters from 25 reflections
*b* = 9.6170 (19) Å	θ = 9–13°
*c* = 14.731 (3) Å	µ = 0.08 mm^−^^1^
β = 101.03 (3)°	*T* = 293 K
*V* = 1044.4 (4) Å^3^	Block, colorless
*Z* = 4	0.30 × 0.20 × 0.10 mm

### Data collection

**Table:**

Enraf–Nonius CAD-4 diffractometer	1045 reflections with *I* > 2σ(*I*)
Radiation source: fine-focus sealed tube	*R*_int_ = 0.011
graphite	θ_max_ = 25.3°, θ_min_ = 2.5°
ω/2θ scans	*h* = −9→8
Absorption correction: ψ scan (North *et al.*, 1968)	*k* = −11→0
*T*_min_ = 0.976, *T*_max_ = 0.992	*l* = 0→17
1971 measured reflections	3 standard reflections every 200 reflections
1897 independent reflections	intensity decay: 1%

### Refinement

**Table:**

Refinement on *F*^2^	Primary atom site location: structure-invariant direct methods
Least-squares matrix: full	Secondary atom site location: difference Fourier map
*R*[*F*^2^ > 2σ(*F*^2^)] = 0.059	Hydrogen site location: inferred from neighbouring sites
*wR*(*F*^2^) = 0.163	H-atom parameters constrained
*S* = 1.00	*w* = 1/[σ^2^(*F*_o_^2^) + (0.069*P*)^2^] where *P* = (*F*_o_^2^ + 2*F*_c_^2^)/3
1897 reflections	(Δ/σ)_max_ < 0.001
136 parameters	Δρ_max_ = 0.14 e Å^−^^3^
0 restraints	Δρ_min_ = −0.14 e Å^−^^3^

### Special details

**Table:**

Geometry. All e.s.d.'s (except the e.s.d. in the dihedral angle between two l.s. planes) are estimated using the full covariance matrix. The cell e.s.d.'s are taken into account individually in the estimation of e.s.d.'s in distances, angles and torsion angles; correlations between e.s.d.'s in cell parameters are only used when they are defined by crystal symmetry. An approximate (isotropic) treatment of cell e.s.d.'s is used for estimating e.s.d.'s involving l.s. planes.
Refinement. Refinement of *F*^2^ against ALL reflections. The weighted *R*-factor *wR* and goodness of fit *S* are based on *F*^2^, conventional *R*-factors *R* are based on *F*, with *F* set to zero for negative *F*^2^. The threshold expression of *F*^2^ > σ(*F*^2^) is used only for calculating *R*-factors(gt) *etc*. and is not relevant to the choice of reflections for refinement. *R*-factors based on *F*^2^ are statistically about twice as large as those based on *F*, and *R*- factors based on ALL data will be even larger.

### Fractional atomic coordinates and isotropic or equivalent isotropic displacement parameters (Å^2^)

**Table:**

	*x*	*y*	*z*	*U*_iso_*/*U*_eq_	
O	0.8616 (3)	−0.2547 (2)	0.13823 (13)	0.0718 (6)	
N	0.7349 (5)	0.1342 (3)	−0.3297 (2)	0.1076 (12)	
C1	0.7327 (4)	0.0786 (3)	−0.2623 (2)	0.0691 (9)	
C2	0.7316 (4)	0.0085 (3)	−0.17484 (17)	0.0565 (7)	
H2A	0.8471	−0.0380	−0.1550	0.068*	
H2B	0.6376	−0.0620	−0.1842	0.068*	
C3	0.6992 (3)	0.1069 (3)	−0.09873 (18)	0.0484 (7)	
C4	0.6435 (4)	0.2412 (3)	−0.1174 (2)	0.0622 (8)	
H4A	0.6228	0.2727	−0.1782	0.075*	
C5	0.6171 (4)	0.3319 (3)	−0.0472 (3)	0.0716 (9)	
H5A	0.5794	0.4227	−0.0613	0.086*	
C6	0.6466 (4)	0.2874 (3)	0.0411 (2)	0.0685 (9)	
H6A	0.6293	0.3486	0.0876	0.082*	
C7	0.7028 (4)	0.1504 (3)	0.06461 (19)	0.0552 (8)	
C8	0.7346 (4)	0.1027 (3)	0.1570 (2)	0.0675 (9)	
H8A	0.7200	0.1635	0.2042	0.081*	
C9	0.7857 (4)	−0.0299 (4)	0.1779 (2)	0.0685 (9)	
H9A	0.8060	−0.0596	0.2391	0.082*	
C10	0.8086 (4)	−0.1236 (3)	0.10760 (19)	0.0564 (7)	
C11	0.7816 (3)	−0.0820 (3)	0.01827 (18)	0.0495 (7)	
H11A	0.7979	−0.1448	−0.0275	0.059*	
C12	0.7285 (3)	0.0571 (3)	−0.00618 (18)	0.0471 (7)	
C13	0.8857 (4)	−0.3548 (3)	0.0708 (2)	0.0723 (9)	
H13A	0.9233	−0.4415	0.1007	0.108*	
H13B	0.9768	−0.3229	0.0380	0.108*	
H13C	0.7733	−0.3677	0.0280	0.108*	

### Atomic displacement parameters (Å^2^)

**Table:**

	*U*^11^	*U*^22^	*U*^33^	*U*^12^	*U*^13^	*U*^23^
O	0.0897 (16)	0.0689 (14)	0.0570 (12)	0.0037 (12)	0.0148 (11)	0.0088 (11)
N	0.177 (4)	0.085 (2)	0.062 (2)	−0.005 (2)	0.024 (2)	0.0025 (17)
C1	0.092 (2)	0.064 (2)	0.0507 (18)	−0.0084 (18)	0.0111 (16)	−0.0041 (16)
C2	0.0636 (19)	0.0525 (17)	0.0549 (17)	−0.0034 (14)	0.0150 (14)	−0.0016 (14)
C3	0.0441 (15)	0.0478 (16)	0.0553 (17)	−0.0072 (13)	0.0142 (13)	−0.0041 (13)
C4	0.065 (2)	0.0532 (18)	0.070 (2)	−0.0012 (15)	0.0164 (15)	0.0030 (16)
C5	0.074 (2)	0.0490 (18)	0.097 (3)	0.0035 (16)	0.0285 (19)	−0.0043 (18)
C6	0.073 (2)	0.057 (2)	0.084 (2)	−0.0069 (16)	0.0352 (18)	−0.0222 (17)
C7	0.0506 (17)	0.0552 (19)	0.0636 (19)	−0.0093 (14)	0.0210 (14)	−0.0135 (15)
C8	0.074 (2)	0.073 (2)	0.062 (2)	−0.0102 (18)	0.0293 (16)	−0.0217 (17)
C9	0.078 (2)	0.082 (2)	0.0499 (17)	−0.0113 (19)	0.0234 (16)	−0.0027 (17)
C10	0.0572 (18)	0.0583 (18)	0.0552 (18)	−0.0043 (14)	0.0141 (14)	0.0006 (15)
C11	0.0491 (16)	0.0508 (17)	0.0519 (17)	−0.0058 (13)	0.0182 (13)	−0.0061 (13)
C12	0.0384 (15)	0.0485 (16)	0.0565 (17)	−0.0098 (12)	0.0142 (12)	−0.0069 (13)
C13	0.078 (2)	0.0579 (19)	0.079 (2)	0.0039 (16)	0.0087 (18)	0.0028 (17)

### Geometric parameters (Å, °)

**Table:**

O—C10	1.372 (3)	C6—H6A	0.9300
O—C13	1.419 (3)	C7—C8	1.413 (4)
N—C1	1.130 (4)	C7—C12	1.417 (3)
C1—C2	1.456 (4)	C8—C9	1.350 (4)
C2—C3	1.522 (3)	C8—H8A	0.9300
C2—H2A	0.9700	C9—C10	1.407 (4)
C2—H2B	0.9700	C9—H9A	0.9300
C3—C4	1.369 (4)	C10—C11	1.353 (4)
C3—C12	1.422 (3)	C11—C12	1.422 (4)
C4—C5	1.396 (4)	C11—H11A	0.9300
C4—H4A	0.9300	C13—H13A	0.9600
C5—C6	1.347 (4)	C13—H13B	0.9600
C5—H5A	0.9300	C13—H13C	0.9600
C6—C7	1.406 (4)		
			
C10—O—C13	117.4 (2)	C8—C7—C12	118.8 (3)
N—C1—C2	179.1 (4)	C9—C8—C7	120.9 (3)
C1—C2—C3	113.2 (2)	C9—C8—H8A	119.5
C1—C2—H2A	108.9	C7—C8—H8A	119.5
C3—C2—H2A	108.9	C8—C9—C10	120.4 (3)
C1—C2—H2B	108.9	C8—C9—H9A	119.8
C3—C2—H2B	108.9	C10—C9—H9A	119.8
H2A—C2—H2B	107.8	C11—C10—O	124.9 (3)
C4—C3—C12	119.7 (3)	C11—C10—C9	120.6 (3)
C4—C3—C2	121.6 (3)	O—C10—C9	114.5 (3)
C12—C3—C2	118.7 (2)	C10—C11—C12	120.5 (3)
C3—C4—C5	121.5 (3)	C10—C11—H11A	119.8
C3—C4—H4A	119.2	C12—C11—H11A	119.8
C5—C4—H4A	119.2	C7—C12—C3	118.3 (3)
C6—C5—C4	119.8 (3)	C7—C12—C11	118.7 (2)
C6—C5—H5A	120.1	C3—C12—C11	123.0 (2)
C4—C5—H5A	120.1	O—C13—H13A	109.5
C5—C6—C7	121.4 (3)	O—C13—H13B	109.5
C5—C6—H6A	119.3	H13A—C13—H13B	109.5
C7—C6—H6A	119.3	O—C13—H13C	109.5
C6—C7—C8	121.9 (3)	H13A—C13—H13C	109.5
C6—C7—C12	119.3 (3)	H13B—C13—H13C	109.5

## References

[bb1] Depreux, P. & Lesieur, D. (1994). *J. Med. Chem.* **37**, 3231–3239. 10.1021/jm00046a006 7932550

[bb2] Enraf–Nonius (1994). *CAD-4 EXPRESS* Enraf–Nonius, Delft, The Netherlands.

[bb3] Harms, K. & Wocadlo, S. (1995). *XCAD4* University of Marburg, Germany.

[bb4] North, A. C. T., Phillips, D. C. & Mathews, F. S. (1968). *Acta Cryst.* A**24**, 351–359.

[bb5] Sheldrick, G. M. (2008). *Acta Cryst.* A**64**, 112–122. 10.1107/S0108767307043930 18156677

[bb6] Spek, A. L. (2009). *Acta Cryst.* D**65**, 148–155. 10.1107/S090744490804362X PMC2631630 19171970

[bb7] Yous, S. & Andrieux, J. (1992). *J. Med. Chem.* **35**, 1484–1486. 10.1021/jm00086a018 1315395

